# Diseases and Injuries of the Ear

**Published:** 1889-06

**Authors:** 


**Diseases and Injuries of the Ear: Their Prevention and Cure.** By CHARLES HENRY BURNETT, A. M., M. D., Aural Surgeon to Presbyterian Hospital; Lecturer on Otology, Women's Medical College of Pennsylvania, in Philadelphia, etc., etc. 12mo, extra, cloth, pp. 154; price, $1.00. Philadelphia: J. B. Lippincott Co. 1889.

This is a most valuable little book. Dr. Burnett has presented his subject, as was his desire, in a form free from technical terms, so that it can be understood by any one. Seldom have we seen a work more fitted for the use of the non-medical reader than the one before us. It should be put into the schools and used as a text-book. In this way it would do an immense amount of service. The older pupils in our high schools would, through it, learn to avoid many of the causes leading to defective hearing. We believe, also, that the teachers could derive benefit from its study. Could more authors be found who would write in the same plain, untechnical way, about other organs of the body, the first step would be taken towards clearing the dense mass of ignorance in which the majority rest in regard to the human organism. This book is divided into three parts. Part I. relates to the Structure and Function of the Ear. Part II. to Common Diseases and Injuries of the Ear: their Prevention and Cure. Part III. to the Aural Hygiene of the Deaf. In each part we have many practical lessons told in same plain-spoken manner. It would be difficult to select one part as more meritorious than another. We repeat that we heartily commend it, and wish for it a wide circulation. The publishers have issued the work as one of the series of "Practical Lessons in Nursing," and have presented it well printed and bound in an attractive manner. The price is low and brings it within the reach of all.

